# Oblique *Corpectomy* to Manage Cervical Myeloradiculopathy

**DOI:** 10.1155/2011/734232

**Published:** 2011-10-19

**Authors:** Chibbaro Salvatore, Makiese Orphee, Bresson Damien, Reiss Alisha, Poczos Pavel, George Bernard

**Affiliations:** ^1^Department of Neurosurgery, Lariboisiere University Hospital, 75475 Paris Cedex 10, France; ^2^Service de Neurochirurgie, Hôpital Lariboisière, 2 rue Ambroise Paré, 75475 Paris cedex 10, France

## Abstract

*Background*. The authors describe a lateral approach to the cervical spine for the management of spondylotic myeloradiculopathy. The rationale for this approach and surgical technique are discussed, as well as the advantages, disadvantages, complications, and pitfalls based on the author's experience over the last two decades. *Methods*. Spondylotic myelo-radiculopathy may be treated via a lateral approach to the cervical spine when there is predominant anterior compression associated with either spine straightening or kyphosis, but without vertebral instability. *Results*. By using a lateral approach, the lateral aspect of the cervical spine and the vertebral artery are easily reached and visualized. Furthermore, the lateral part of the affected intervertebral disc(s), uncovertebral joint(s), vertebral body(ies), and posterior longitudinal ligament can be removed as needed to decompress nerve root(s) and/or the spinal cord. *Conclusion*. Multilevel cervical oblique corpectomy and/or lateral foraminotomy allow wide decompression of nervous structures, while maintaining optimal stability and physiological motion of the cervical spine.

## 1. Introduction

Cervical spondylotic myelopathy (CSM) and cervical spondylotic radiculopathy (CSR) are classically approached by anterior single or multiple disc space decompression [1, 2], multilevel corpectomy [3], laminectomy [4–10], or laminoplasty [11–18]. More recently, techniques using lateral multiple oblique *corpectomy* (MOC) and/or foraminotomy [19–28] have been used with increasing frequency. In general, when three or more levels are affected, the preferred techniques remain either an anterior multilevel corpectomy or a posterior route such as laminectomy, open door laminoplasty, and posterior foraminotomy. However, the best management of such pathology (especially if 3 or more levels are involved) remains controversial. The authors consider the cervical spine lateral approach a valid and safe option to treat such pathologies as it provides very good clinical results and maintains long-term spinal stability. The goal of this paper is to further and critically present the idea and rationale of the cervical spine lateral approach with its advantages, disadvantages, complications, and pitfalls in a critical review of their last 2 decades experience.

## 2. Technique Indication

Predominant anterior compression associated with either straightening or kyphosis of the cervical spine in the absence of instability is the general indication for the proposed technique. In cases of both anterior and posterior compression, the posterior approach is probably the best choice as long as cervical lordosis remains preserved. It is very rare that spinal cord compression necessitates both anterior and posterior decompression.

## 3. Inclusion-Exclusion Criteria

### 3.1. Inclusion

- Clinical evidence of cervical myelopathy and/or radiculopathy.
- Cervical CT-MRI scan evidence of single/multiple level nerve roots and/or spinal cord compression, mainly anterolateral and/or myelopathy.
- Evidence of neutral or kyphotic cervical alignment in the lateral cervical plain X-ray, as well as absence of instability documented by cervical dynamic X-ray.

### 3.2. Exclusion

- Soft disc herniation documented with MRI within 6 months (only for MOC).
- Presence of preoperative anterolisthesis >2 mm between any two contiguous vertebral bodies.

## 4. Surgical Technique

### 4.1. Principles

The technique has already been described and reported by the authors [19–27, 29]. The idea and rationale of the present technique is based on the evidence that anterolateral compression of the cervical spine and nerve roots may be best managed by an anterolateral approach because it provides direct exposure of the abnormal area. The described technique is a variation of the Verbiest technique [30–33]. Experience with this technique began in 1989, with the senior author studying the approach on cadaveric specimens. Initially, the anterolateral approach was only employed for foraminotomies to treat severe nerve root compression. Over time and with greater visualization and dexterity, more complex cases were planned. The first oblique corpectomies were completed in 1992. Ultimately, this approach has become routine in our department for the treatment of spondylotic radiculopathy or myelopathy [19–27, 29]. Preoperative planning and imaging are critical. The decision of which side to perform the procedure is first based on the clinical picture and which side is most symptomatic. If symptoms are bilateral, the side with the larger osteophytes or disc herniation is chosen. If either clinically or radiologically there is no predominant side, the approach chosen is on the side of the smaller vertebral artery (VA). The idea and rationale are that it is easier and safer when the artery is clearly visualized and under control. 

#### 4.1.1. Patient Positioning and Exposure

**Figure 1:**
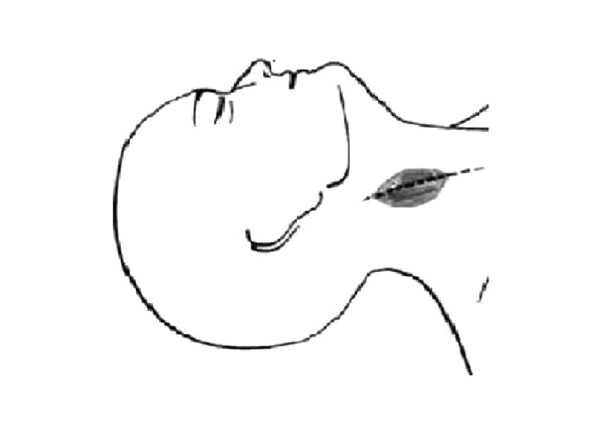
Skin incision and head position.

**Figure 2:**
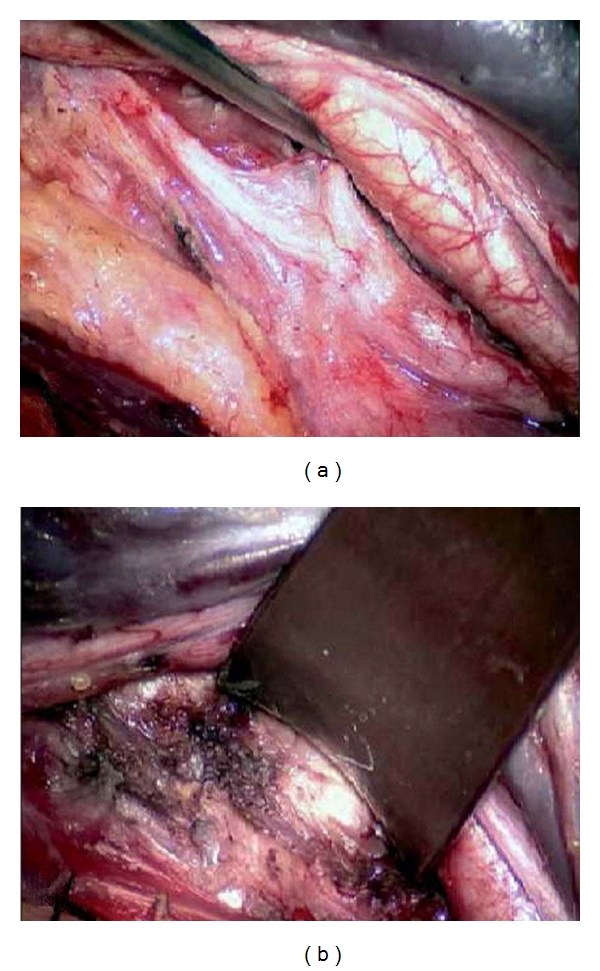
(a, b) Opening the field between the internal jugular vein and the sternocleidomastoid muscle with the fat sheath in the depth.

The patient is positioned supine, with the head slightly extended and rotated to the contralateral side. A longitudinal skin incision is made along the medial border of the sternocleidomastoid (SCM) muscle at the level of the vertebral bodies to be exposed (see also Figure 1). The incision may extend to the mastoid tip to expose C2-3 and to the sternal notch to expose C7-T1. The subcutaneous tissue and the platysma muscle are incised along with the skin incision. The natural space between the SCM muscle and the internal jugular vein is opened by sharp dissection (see also Figures 2(a) and 2(b)). The SCM muscle is retracted laterally, while the great vessels, trachea, and esophagus are kept undissected medially and protected by a blunt retractor. There is always a variable amount of fat in the depth of this space. This fatty sheath surrounds the accessory nerve, which must be identified when the C2-C3 and the C3-C4 levels need to be exposed. At this point, the transverse processes can be easily palpated and then visually identified. The transverse processes are covered by the prevertebral muscles. Under the aponeurosis of the longus colli muscle, the sympathetic chain must be recognized. The aponeurosis is divided longitudinally, medial to the sympathetic chain; both the aponeurosis and the sympathetic chain are then retracted laterally. The longus colli muscle is divided along the transverse processes and vertebral bodies at the decided levels and then retracted away from the field. Care must be taken to be sure the VA is not entering the transverse foramen at an abnormally high level (C5, C4, or even C3); in this case, the artery is running before the transverse processes and may be injured during the longus colli muscle division. At this point, the transverse processes and the lateral aspect of the vertebral bodies are clearly exposed.

#### 4.1.2. Foraminotomy

The intervertebral foramen is opened by removing the anterior part of the transverse foramen with a Kerrison Rongeur after it is identified via subperiosteal dissection; this manoeuvre helps with additional lateral VA mobilization by creating a plane between the lateral aspect of the uncovertebral joint and the medial border of the VA. Once both structures are separated, the hypertrophied uncovertebral joint can be safely removed with a drill and/or rongeurs. In this way, the cervical nerve root can be completely decompressed from its dural origin up to the VA lateral border.

#### 4.1.3. Oblique Corpectomy

**Figure 3:**
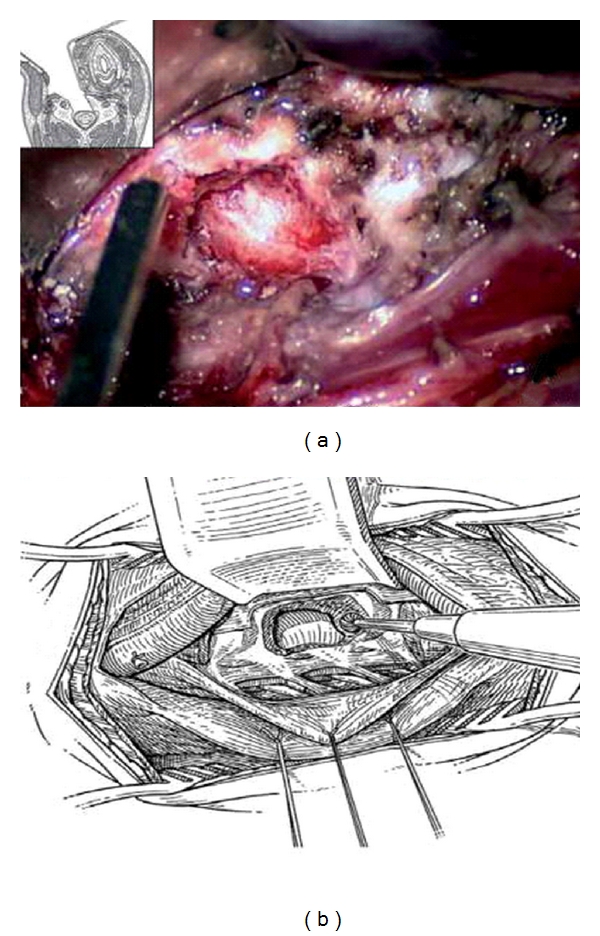
(a, b) Resection of lateral aspect of the vertebral body. (b) Schematic drawing.

**Figure 4:**
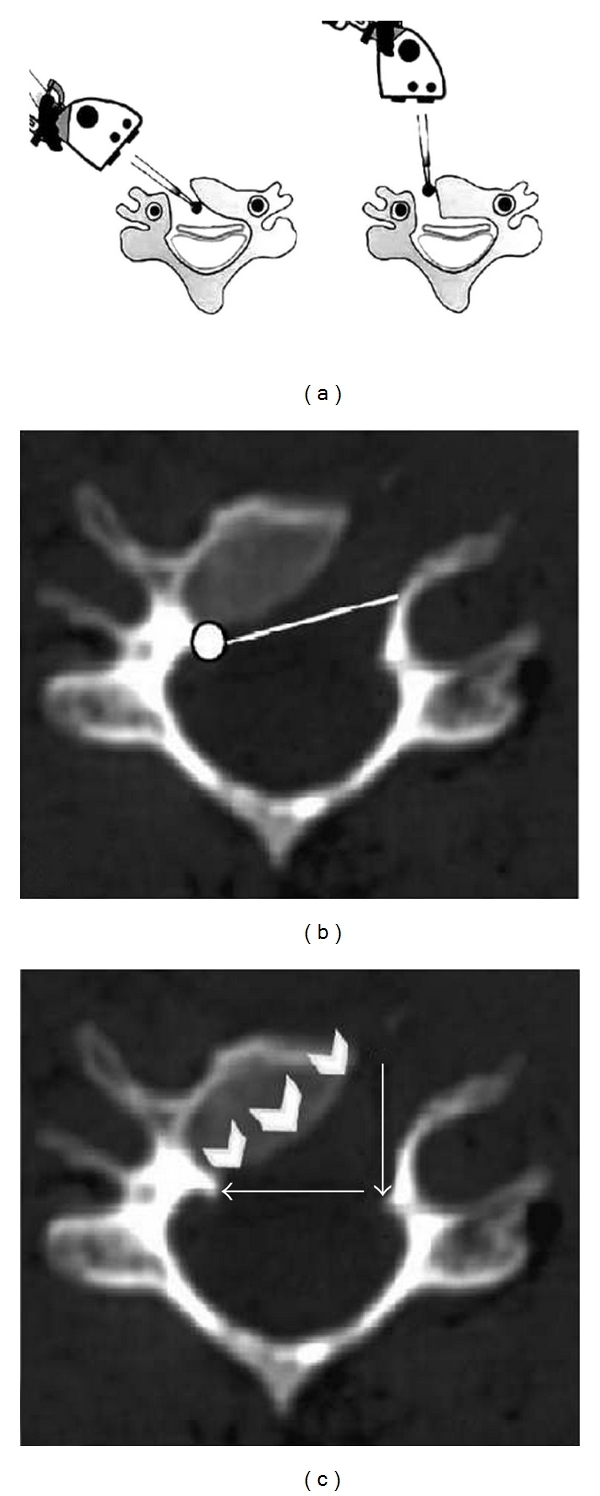
(a) The microscope is first positioned vertically (right picture). The drilling begins vertically, up to the posterior cortical bone, at that time the microscope has to be repositioned to get an oblique view (left picture). The drilling is then continued horizontally up to the contralateral side. (b)-(c) Postoperative axial CT scan showing the directions (white arrows) and final area of drilling.

**Figure 5:**
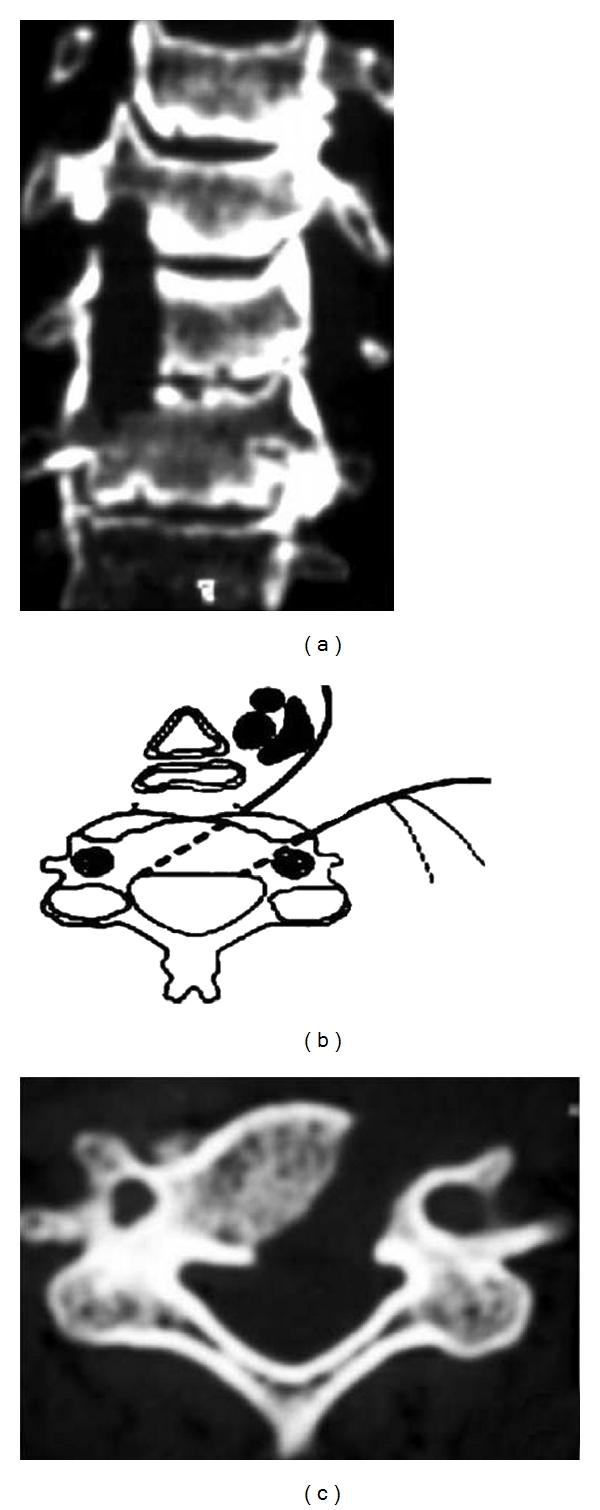
Postoperative (a) coronal and (c) axial CT scan showing wide spinal canal decompression after 1 level MOC; (b)— schematic drawing.

**Figure 6:**
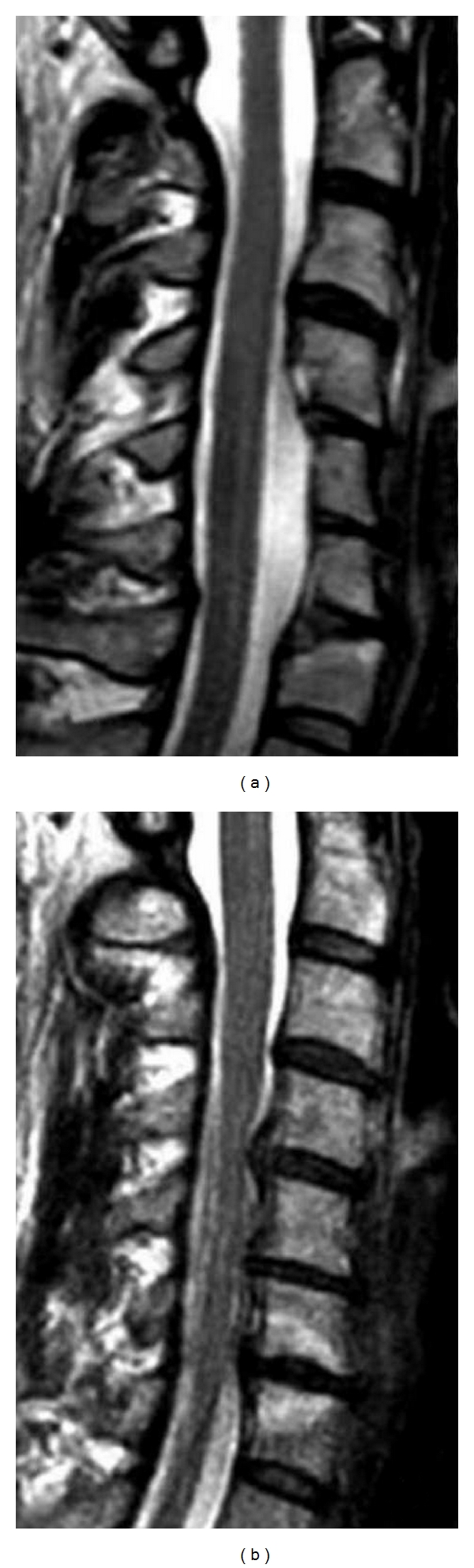
(b) Preoperative T2-weighted sagittal MRI showing spinal cord compression at the C4-C5, C5-C6, and C6-C7 levels. (a) Postoperative T2-weighted sagittal MRI illustrating the wide spinal cord decompression after 2 levels MOC.

After radiological identification of the correct level, we start the corpectomy, using a cutting drill, on the bodies on both sides of the disc. We keep the direction of the drill parallel to the endplate; the corpectomy continues until the cortical bone of the posterior aspect of the body is found. The pieces of bone and disc remaining in between are then removed. Next, the drilling is extended obliquely toward the opposite side. It is very important to start with a vertical trench just medial to the VA and then to move obliquely so as to reduce, as much as possible, the extent of bone resection (see also Figures 3(a) and 3(b), Figures 4(a), 4(b), and 4(c)). The intervertebral discs are incised and removed up to the posterior margin of vertebral body. At this stage, the vertebral body is drilled obliquely from the lateral side toward the opposite posterolateral corner. More than half of the vertebral body is preserved creating a convex-shaped posterior aspect. Next, drilling is turned towards a point of the posterior aspect of the vertebral body, which has been precisely located on the preoperative computed tomographic (CT) scan. It is located at the limits of the osteophytes and often corresponds to the junction between the body and the opposite pedicle (see also Figure 5(a), 5(b), and 5(c) and Figures 6(a)-6(b)).

#### 4.1.4. Posterior Longitudinal Ligament Resection

Next, the posterior longitudinal ligament must be opened longitudinally and as much as possible removed to ensure that optimal cord decompression has been obtained.

### 4.2. Advantages of the Technique

The present procedure provides the following advantages:

1. Wide anterolateral decompression of the spinal canal and foramen at single or multiple levels (multiple levels to decompress is not a limitation).
2. Easy access to any level including the upper ones (C2-C3, C3-C4).
3. Kyphotic change is not a contraindication, as long as vertebral stability is preserved.
4. There is no need for bone grafting and/or instrumentation making the technique very suitable for elderly people and heavy smokers.
5. The lateral approach, by using a different path, when compared to the standard anterior approach, offers an excellent visual alternative as the field between the SCM muscle and the internal jugular vein is opened. This is particularly desirable in cases of recurrence after previous anterior surgery, because there is no need to mobilize tedious postoperative scar tissue.
6. The horizontal drilling is safer.

### 4.3. Disadvantages of the Technique

The present procedure has the following disadvantages:

1. Bilateral radiculopathy may not be treated in a single-staged procedure; in these cases, an anterior midline approach remains the procedure of choice.
2. In cases where delayed contralateral radiculopathy appears, then selective microsurgical nerve root decompression may be advocated; although, we suggest spine stabilization/fusion.
3. The anterolateral technique, by exposing multiple anatomical critical structures, is a procedure with a steep learning curve. Good knowledge of  VA anatomy and its possible variations is essential. However, surgeons should not be discouraged by initial difficulties and should keep in mind that after having performed the procedure 10 times, the operative time will be substantially reduced.
4. Stretch and potential damage of XI nerve (when exposing C2-C3 level), and Horner's syndrome (when approaching C4-C7) are well known but rare complications.
5. Kyphosis, if present, is not corrected by the technique.

### 4.4. Clinical Experience

Since 1989, we have completed 499 procedures using this technique. Analysis of our experience allows the following considerations: at a mean followup of 111 months (range 9 to 202 months), a global recovery rate of 87.6% was recorded for CSM using the following formula:

(1) $$\begin{array}{cc} & \text{recovery}\;\text{rate}\;\left(\%\right)\\ & \;\;=\frac{\left[\text{postop}\;\text{mJOA}\;\text{score}-\text{preop}\;\text{mJOA}\;\text{score}\right]\times 100}{17-\text{preop}\;\text{mJOA}\;\text{score}} \end{array},$$

and a global recovery rate of 95% for CSR using a score obtained by multiplying the intensity (VAS scale 0–10) and the duration scores, ranging in this way, from 0 to 100.

The cervical lateral approach for CSM and/or CSR can be extended to as many levels as required, and the number of levels is not considered a limit for this procedure. A total of 900 levels were decompressed in 499 patients (C2-C3 in 35 cases, C3-C4 in 122, C4-C5 in 188, C5-C6 in 296, C6-C7 in 128, and C7-T1 in 9). Oblique vertebrectomy was performed at one level in 221 patients, two levels in 121 patients, three levels in 88 patients, four levels in 32 patients, and five levels in 9 patients. 

The mean operation time was 118 minutes (range 73 to 183 minutes); mean intraoperative estimated blood loss was 68 mL (range 28 to 280 mL) and the mean hospitalization time was 6 days (range 2 to 14 days).

Only three patients (less than 1%) required delayed stabilization. The first developed a disc herniation; the second had an unrecognized congenital bone malformation; the third developed segmental instability at a level above the treated levels.

No cerebrospinal fluid leakage (0%), infections (0%), nor C5 deficit and/or dysphagia/dysphonia were observed in our series. A transient HS was observed in 14 patients (3%); in almost all of them, symptoms markedly resolved within 3 months with less than 1% (4 cases) retaining permanent impairment.

## 5. Discussion

The lateral foraminotomy and the oblique *corpectomy* technique, by preserving over 50% of the vertebral body and preserving two of the three columns, do not compromise spinal stability so that bone grafts or instrumental arthrodesis are not necessary [22]. Patient selection for these procedures is crucial; for this reason all patients with clear spine instability (slippage >2 mm between at 2 adjacent vertebral bodies on dynamic X-ray) and/or with a preoperative fixed listhesis 2 mm were excluded. In our series, only three patients required delayed stabilization, and aside from these three patients, a change in the spinal curvature of more than 5 degrees was never observed postoperatively at the level of the surgical decompression, regardless of the preoperative spinal curvature.

The lateral approach [19, 20, 22–29] differs substantially from other anterolateral approaches as it leads directly to the lateral aspect of the vertebral body and the transverse process which are covered by the prevertebral muscles. When dividing the prevertebral muscles, it is important to identify and preserve the sympathetic chain running under the aponeurosis. Manipulation of the sympathetic nerves may result in a postoperative Horner's syndrome (HS), but it is mild and transient if the main trunk of the sympathetic chain is preserved. In our series, three percent of patients experienced a transient HS as a consequence of manipulation of the sympathetic nerves. In almost all cases, symptoms markedly resolved within 3 months with less than 1% (4 cases) retaining permanent impairment. The majority of HS cases (9 cases) occurred in the first 3 years of our practice. Horner's syndrome can occur and constitutes a disadvantage of the technique, but as demonstrated by this series, incidence decreases significantly with increasing experience. In our experience, HS is almost always temporary, if careful identification and gentle retraction without dissection of the sympathetic chain is performed. We do not agree with other authors, Rocchi et al. [28], who have proposed dissection of the sympathetic chain to avoid its functional damage. 

Similarly, when exposing levels above C3, the accessory nerve must be retracted as gently as possible and kept protected by a fat pad around it. Morbidity resulting from the dissection of the accessory nerve is very unlikely and never occurred in our experience. 

With the lateral approach, controlled, but not mobilized, of the VA provides protection to surrounding important structures. Direct visualization of the VA allows for safe drilling of the posterolateral corner of the vertebral body and the control of the distal nerve root. Troublesome venous bleeding from the perivertebral venous plexus can be prevented by preservation of the periosteal sheath around the VA. Furthermore, care must be taken to identify any abnormal course of the VA, especially entry of the VA into the transverse canal at an abnormally high level, which may be the C5, C4, or even C3 level [21]; this can be easily achieved by carefully examining a standard preoperative MRI and/or MRA. 

Practical pearls offered by the authors while performing this technique include the placement of the suction device in front of the VA to provide protection in the case of inadvertent sliding during drilling. Also with the oblique corpectomy, there is a natural tendency to drill bone inadvertently and unnecessarily in a horizontal plane which may compromise spinal stability; therefore, the operative microscope should be set obliquely (to have an oblique view) to avoid this tendency. Practically, it is very important to start with a vertical trench just medial to the VA and then to move horizontally, reducing in this way, as much as possible, the extent of bone resection (see also Figure 4(a)); this represents the first surgical pitfall. Another problem is represented by the absence of an anatomic landmark to define where the horizontal drilling should be stopped; this constitutes the second pitfall, and to solve it we determine on the preoperative CT scan the extent of drilling. In general, the limits of the osteophytes set the length of bone drilling and, very often, correspond to the junction between the body and the opposite pedicle. Verifying the adequacy of the decompression in the horizontal plane is important to the success of this procedure. Therefore, the distance between the contralateral pedicle and the medial border of the ipsilateral VA is measured on the preoperative CT scan, or as more recently by using the perioperative IGS (image-guided system) which is very reliable and effective also recently confirmed by Lee et al. [34]. In the majority of cases, the distance varies between 22 and 28 mm. In our series, no cerebrospinal fluid leakage, infections, C5 deficits/or dysphagia/dysphonia were observed. We have no clear explanation as to why these complications were not encountered; they may be related to the absence of any distractions during the procedure. As for the lack of C5 deficits postoperatively, there is no clear explanation, but it could be related to this approach and the better visualization for the surgeon of the cervical spinal root at the foramen. Since this technique does not require medial traction, the trachea and the esophagus are barely touched by holding a hand blade (self-retaining retractors are never used). Over last few years various clinical series [34–39] about oblique corpectomy as well as experimental studies [40, 41] have been published; the formers have shown, according to our experience, the effectiveness and reliability of such technique in managing cervical myeloradiculopathy; in all reports the authors agreed about the related good functional outcome as well as on the preservation of spinal stability with its physiological motion which represent a considerable advantage in the treatment of such pathology. 

Finally, we would like to emphasize that this approach is initially a demanding procedure, and the learning curve may be long for some. In the authors' experience, it is sufficient to perform the procedure few times in the cadaver laboratory, and for the first few times, to be assisted by an experienced surgeon. We also would like to stress that good knowledge of VA anatomy and its variations is essential to performing this operation and that careful analysis of preoperative imaging is crucial.

## 6. Conclusions

Although multilevel oblique corpectomy and/or simple foraminotomy via a lateral approach remains a rather demanding technique with a substantial learning curve, we believe it is a valid alternative for the management of multisegmental cervical spondylosis. Good knowledge of VA variations is essential and careful analysis of preoperative imaging is mandatory. This technique does not compromise stability, as much as anterior approaches do. The incidence of early and late postoperative complications is lower, and bone grafting is not necessary, allowing for it to be used in patients with a low fusion rate such as the elderly, diabetics, and heavy smokers. It also permits early patient mobilization with no postoperative immobilization. As often is the case, optimal results rely on scrupulous selection of patients and preservation of cervical spine stability.
